# Combining Low Temperature Fluorescence DNA-Hybridization, Immunostaining, and Super-Resolution Localization Microscopy for Nano-Structure Analysis of ALU Elements and Their Influence on Chromatin Structure

**DOI:** 10.3390/ijms18051005

**Published:** 2017-05-07

**Authors:** Matthias Krufczik, Aaron Sievers, Annkathrin Hausmann, Jin-Ho Lee, Georg Hildenbrand, Wladimir Schaufler, Michael Hausmann

**Affiliations:** 1 Kirchhoff-Institute for Physics, Heidelberg University, Im Neuenheimer Feld 227, 69120 Heidelberg, Germany; krufczik@kip.uni-heidelberg.de (M.K.); Sievers_Aaron@web.de (A.S.); annkathrin.hausmann@googlemail.com (A.H.); jin-ho.lee@kip.uni-heidelberg.de (J.-H.L.); hilden@kip.uni-heidelberg.de (G.H.); 2 Department of Radiation Oncology, University Medical Center Mannheim, Heidelberg University, Theodor-Kutzer-Ufer 3-5, 68159 Mannheim, Germany; 3 German Cancer Research Center (DKFZ), Im Neuenheimer Feld 280, 69120 Heidelberg, Germany; w.schaufler@dkfz.de

**Keywords:** combinatorial oligonucleotide FISH (COMBO-FISH), combined immuno-staining, cell nucleus, chromatin arrangement, localization microscopy, ALU-repeats, low LET radiation exposure

## Abstract

Immunostaining and fluorescence in situ hybridization (FISH) are well established methods for specific labelling of chromatin in the cell nucleus. COMBO-FISH (combinatorial oligonucleotide fluorescence in situ hybridization) is a FISH method using computer designed oligonucleotide probes specifically co-localizing at given target sites. In combination with super resolution microscopy which achieves spatial resolution far beyond the Abbe Limit, it allows new insights into the nano-scaled structure and organization of the chromatin of the nucleus. To avoid nano-structural changes of the chromatin, the COMBO-FISH labelling protocol was optimized omitting heat treatment for denaturation of the target. As an example, this protocol was applied to ALU elements—dispersed short stretches of DNA which appear in different kinds in large numbers in primate genomes. These ALU elements seem to be involved in gene regulation, genomic diversity, disease induction, DNA repair, etc. By computer search, we developed a unique COMBO-FISH probe which specifically binds to ALU consensus elements and combined this DNA–DNA labelling procedure with heterochromatin immunostainings in formaldehyde-fixed cell specimens. By localization microscopy, the chromatin network-like arrangements of ALU oligonucleotide repeats and heterochromatin antibody labelling sites were simultaneously visualized and quantified. This novel approach which simultaneously combines COMBO-FISH and immunostaining was applied to chromatin analysis on the nanoscale after low-linear-energy-transfer (LET) radiation exposure at different doses. Dose-correlated curves were obtained from the amount of ALU representing signals, and the chromatin re-arrangements during DNA repair after irradiation were quantitatively studied on the nano-scale. Beyond applications in radiation research, the labelling strategy of immunostaining and COMBO-FISH with localization microscopy will also offer new potentials for analyses of subcellular elements in combination with other specific chromatin targets.

## 1. Introduction

Fluorescence in situ hybridization (FISH) using target-specific DNA probes as well as immunostaining using specific antibodies have each become routine techniques in modern molecular biology, cell research, and medical diagnosis [1]. Experiments combining both techniques simultaneously in combination with high-resolution microscopy (a review about high-resolution microscopy is presented in [2,3]) are still challenging because of the different protocol essentials and their influence on the examined structure [4]. FISH of chromatin usually needs DNA denaturation at high temperatures [5], which should be avoided for analysis of DNA structure with high-resolution microscopy. Mongelard et al. [6] avowed the denaturation step of the target DNA as the most damaging step in the FISH procedures. This destructive effect due to heat treatment was clearly shown by super-resolution scanning near-field optical microscopy (SNOM) [7]. In contrast to DNA–DNA FISH of chromatin in cell nuclei mRNA FISH using appropriate oligonucleotides can be optimally performed at nearly physiological temperatures [8] and applied to super-resolution microscopy [8,9] together with immunostaining. Although the combination of DNA-FISH and immunostaining resulted in apparently well-maintained chromatin morphology [4], it should be considered that the usually preferred conventional preparation techniques in super-resolution microscopy determine the practical limits of application [10]. Fornasiero et al. [11] presented an overview of the advantages and disadvantages of the high-resolution microscopy methods regarding various biological tasks. A challenge that must not be underestimated in this context of circumventing possible limits is the development of adequate computer-based analysis methods for the evaluation of the high-resolution data.

In this work, we present a method to analyze the chromatin organization of ALU elements and its heterochromatic environment in intact cell nuclei using an optimized low-temperature DNA-hybridization-protocol for chromatin FISH in combination with immunostaining, super-resolution localization microscopy, and in-house-developed computer-based methods for structural analysis.

In biomedical research and diagnostics, DNA-FISH is routinely used for the detection of numerical and structural chromosome aberrations like gene copy number changes, translocations, deletions, and amplifications in cytological smear preparations, histological sections, and isolated peripheral blood and bone marrow cells [12]. Usually FISH probes manufactured as BAC or YAC (bacterial/yeast artificial chromosome) probes have a length of some ten up to some hundred kilo bases. For specific targeting, all these established protocols require harsh steps of target denaturation by heat treatment accompanied by chaotropic agents [13,14]. This specimen preparation not only has an influence on the nano-structure of the DNA, but seems to be incompatible with the requirements of immunostaining protocols. In so-called low-temperature FISH experiments, thermal denaturation of the target DNA has been omitted and measurements with scanning near-field optical microscopy (SNOM) have shown persuasive results regarding the preservation of metaphase chromosome morphology by renouncement of denaturation [7]. Based on these low-temperature protocols, a novel technique called combinatorial oligonucleotide (COMBO)-FISH was established and proven by Hausmann et al. [15] (for review, see [16]). Oligonucleotide sets with a length of 15 to 30 bases per probe are used [17] that specifically bind within a designated genomic region. Using home-developed algorithms for genome data bank analysis, a computer search for appropriate unique or different oligonucleotides which co-localize in the desired region(s) [18] can result in a specific label and produce a detectable signal in fluorescence microscopy. Compared to standard FISH probes of several kb of DNA, the small COMBO-FISH probes of about 20 bases only are closely attached to the labelled DNA strand, and the fluorochromes have a very small distance to the target (about 20 nm linker length). In combination with localization microscopy [19,20], it is possible to get detailed information about the structure of the stained target [21,22].

COMBO-FISH oligonucleotide probes can be developed and synthesized for any given target (e.g., centromeres, genes, breakpoint regions, trinucleotide expansion repeats, etc.), if the species has been sequenced completely for any given organism. Depending on the target and type of oligonucleotide, the probe binding can be realized by Watson–Crick or Hoogsteen bonding. The latter requires either pure purine or pure pyrimidine oligonucleotide probes, and results in triple strand binding. DNA denaturation of the target strand by heat treatment can be avoided [15,23]. In this case, the structural influence on the chromatin is negligibly low.

An ALU element is a short stretch of DNA (SINE = short interspersed nuclear element) which appears in different kinds and repetition rates in large numbers in primate genomes and other species. Thirty-seven different ALU sequence families are known [24]. They differ in their lengths depending on their evolutionary age, but show a close similarity which can be expressed by the probability of a consensus-sequence in the different families [25]. These ALU elements seem to be involved in gene regulation, genomic diversity [26], disease [24] and DNA repair [27]. Much work on ALUs is focused on the sequence information, but for the understanding of regulatory mechanisms, it may also be necessary to study the organization, structure, and distribution of ALU elements in the nucleus to elucidate their role for chromatin modelling, functionally correlated structure formation, and other effects. Using chromosome conformation capture techniques like Hi-C [28], mega-base-sized chromatin-interaction domains were found and seem to be a structural feature of the genome organization. ALU elements seem to be enriched at boundary regions of chromosome interaction domains, indicating a role in the spatial organization of the genome [29,30]. In addition, ALU elements are embedded in prominent oncogenes (e.g., *BRCA1*) [31]. During evolution, ALU elements as transposonable elements seem to play a prominent role in genome adaptation to environmental stress [32], resulting in many different functions currently known [33]. The “network-like” organization of ALU-elements over the whole genome makes them an ideal candidate for the description of genome-wide topological changes induced by environmental pressure such as radiation treatment.

Localization microscopy has been realized in several different forms [3], such as photoactivated localization microscopy (PALM) [34], fluorescence photoactivation localization microscopy (F)PALM [35], stochastic optical reconstruction microscopy (STORM) [36,37], direct stochastic optical reconstruction microscopy (dSTORM) [38], ground state depletion microscopy followed by individual molecule return (GSDIM) [39], or super-resolution optical fluctuation imaging (SOFI) [40], etc. As a technique of single molecule imaging, it gets full benefit from COMBO-FISH, which can be used for nano-probing with single dye molecules [21,22]. Implementation of COMBO-FISH probes into a global cell nucleus network like heterochromatin can be applied to get additional structural information about certain regions of the nucleus in the frame of the chromatin network. Localization microscopy uses optical isolation of the fluorophores to circumvent the Abbe diffraction limit of optical resolution. Differently colored or switchable fluorophores can be used to achieve optical isolation. SPDM (“spectral position determination microscopy”) [19]—one type of localization microscopy—uses a property of many fluorophores [41]: During illumination with high power lasers (kW/cm^2^), these fluorophores are reversibly bleached. With a certain probability, they recover from the bleached state to become fluorescent again. The stochastic recovery of fluorophores from the dark state yields to an optical isolation by “blinking”, which allows a precise position determination far below the Abbe limit. Acquisition of a high number of images over time results in a good reconstruction of the original distribution of fluorescent markers [42].

The raw data for a localization image consist of a large number of images in an image stack acquired in a time series. According to the mentioned principle of localization microscopy, on every image of the image stack, only a few fluorophores are fluorescently switched on. This is used to compute the exact positions of the fluorophore. A point signal is imaged by a microscope into an Airy disc according to the point-spread function (PSF). In practice, the intensity of the maximum of this PSF must be at least four times higher than the background intensity to get registered as an event. The area of increased intensity must fit into a square region of interest (ROI) because the PSF has a round shape. The double of the background intensity is subtracted from each pixel of the ROI. The resulting reduced ROI is effectively rounded. The center of gravity *µ* and the associated standard deviation *σ* of the signal are determined using two-dimensional Gaussian function *f*. From this, the localization precision Δ*µ* can be calculated, which depends among others things (compare formulas below), on the number of detected photons *q_i_*, and the intensity background *N_B_*. A more detailed description can be found in [43].

$$f(\vec{x})=\frac{Q}{2\pi \sigma_{x}\sigma_{y}}e^{-\frac{{\left(\frac{x\;-\;\mu_{x}}{\sigma_{x}}\right)}^{2}+\;{\left(\frac{y\;-\;\mu_{y}}{\sigma_{y}}\right)}^{2}}{2}}$$

$$Q=\sum q_{i}\;\vec{\mu }=\sum \frac{q_{i}}{Q}{\vec{x}}_{i}\;\sigma_{x}^{2}=\sum {\left(x_{i}-\mu_{x}\right)}^{2}\frac{q_{i}}{Q}$$

$$\Delta \mu_{x}=\sqrt{\frac{1}{12Q}+\sum {\left(\frac{x_{i}-\mu_{x}}{Q}\right)}^{2}(q_{i}+N_{B})}$$

**Figure 1:**
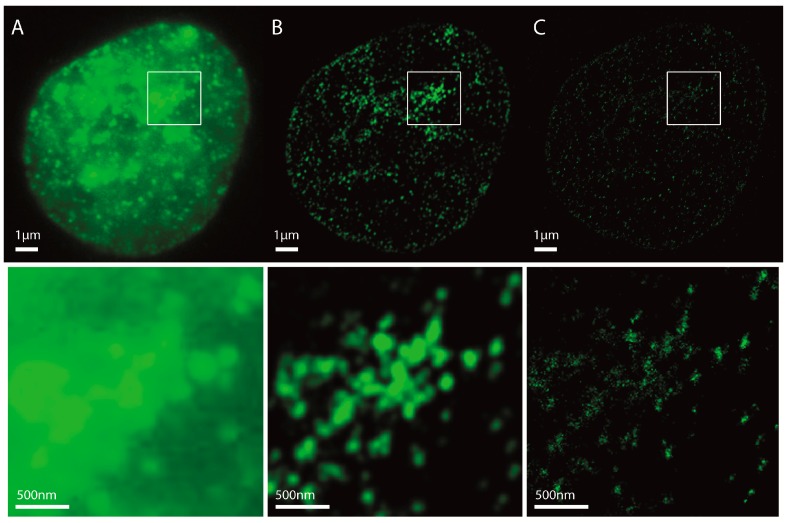
Localization microscopy images of H3K9me3 immunostaining in a SkBr3 cell nucleus. (**A**) Conventional widefield microscopy image; (**B**) Density image. The image is created from the coordinate matrix. For this purpose, the localization data stored in nanometres in the coordinate matrix must be converted into pixels. For this, a pixel size in nm must be determined. The coordinates from the matrix are then assigned to the corresponding pixels. Then, in a fixed radius *R* around each coordinate, it is determined how many further coordinates are within this radius. This value is assigned to the coordinate. In order to emphasize contiguous structures, each coordinate with an assigned value greater than zero is the starting point of a Gaussian distribution with a given *σ*. The sum of all Gaussian distributions then represents the intensity distribution of the Gaussian-filtered density image (example: Pixel size = 10 nm/pixel, radius *R* = 1000 nm, Gaussian filter *σ* = 50 nm); (**C**) Standard localization image. This image is also created from the localization data. Again, a pixel size must be determined. Here, every coordinate is the starting point of one Gaussian distribution with the localization precision as *σ*. (pixel size = 10 nm/pixel).

This results in a matrix which contains the coordinates for every measured fluorophore with a typical localization precision of about 10 nm [44]. The pointillist localization image can be used for further analyses based on distance and frequency measurements (e.g., [45,46]). Usually, density images (see Figure 1 for the different possible methods to produce images from the localization data) are used. The images represent a form of visualization, and due to clarity of structure recognition, do not always require highest resolution. Nevertheless, the full resolution is used for further quantification of nano-structure analyses like cluster formation, etc. [46,47].

## 2. Results

### 2.1. Localization Microscopy after Immunohistochemistry Labelling

Localization microscopy data result in a matrix of coordinates of the detected signals. This list also contains a set of other data, such as intensity, localization precision, errors of the values, etc. By means of pointillist visualization (for details of representation, see Figure 1) of the detected labelling molecule loci, localization microscopy offers an optical image scaled by the applied pixel size. Compared to imaging using the same setup in a conventional wide-field mode, the resolution is much better. Better resolution means that the minimal detectable point distance is considerably smaller as compared to conventional optical far-field microscopy. Localization microscopy can optically distinguish fluorophores with a minimum distance on the order of 10 nm. On the other hand, conventional microscopy requires a minimum distance of about 200 nm for the separation of two points. Figure 1 shows localization images of H3K9me3 immunostaining in comparison to conventional microscopy. Here, H3K9me3 was used as a heterochromatin marker [48], especially due to its staining efficiency.

### 2.2. Probe Design for ALU-Combinatorial Oligonucleotide (COMBO)-Fluorescence In Situ Hybridization (FISH)-Staining

For the sequence of the ALU COMBO-FISH oligonucleotide probe used, genome data base studies were performed. The typical ALU consensus sequence is about 300 bp long (5’-3’) [25]: GGCCGGGCGCGGTGGCTCACGCCTG***TAATCCCAGCACTTTGG***GAGGCCGAGGCGGGCGGATCACCTGAGGTCAGGAGTTCGAGACCAGCCTGGCCAACATGGTGAAACCCCGTCTCTACTAAAAATACAAAAATTAGCCGGGGCGTGGTGGCGCGCGCCTGTAATCCCAGCTACTCGGGAGGCTGAGGCAGGAGAATCGCTTGAACCCGGGAGGCGGAGGTTGCAGTGAGCCGAGATCGCGCCACTGCACTCCAGCCTGGGCGACAGAGCGAGACTCCGTCTC.

**Figure 2:**
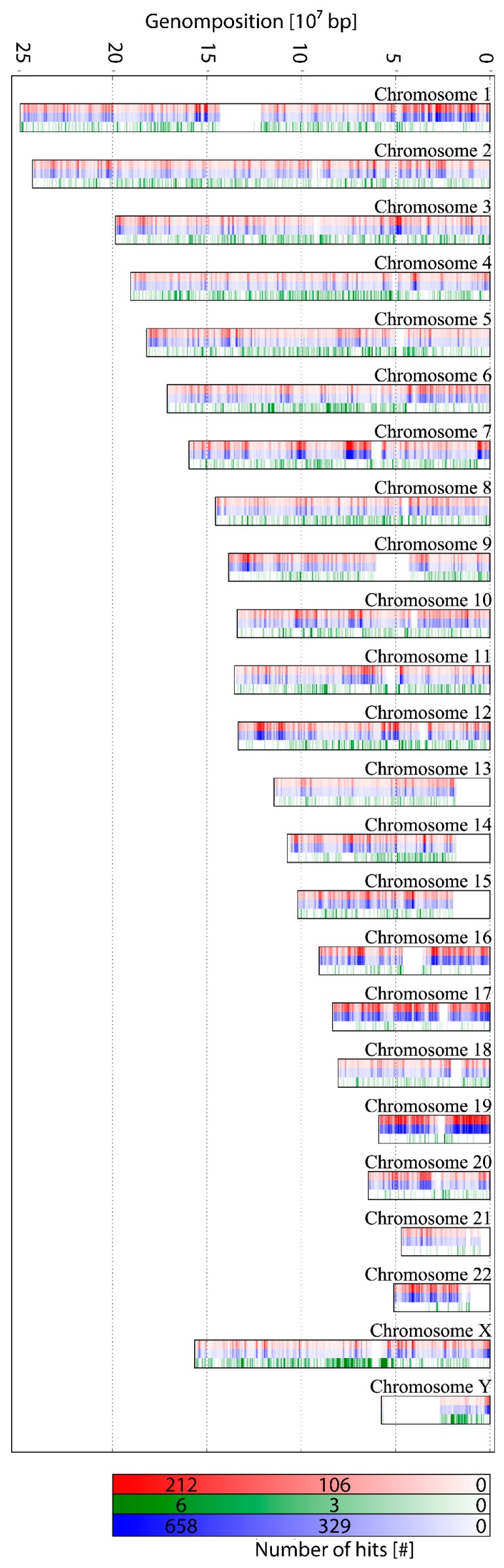
ALU-distribution along the genome: The intensity of the bars indicates the frequency within a 500 kb section of the given chromosome. Red: Position of the designed 17mer ALU probe. The sequence associated with the ALU probe appears in the entire genome at different frequency densities. Blue: Corresponding positions of the ALU consensus sequence. The number of emergence of the 17mer probe sequence was compared with the density of the ALU consensus sequence by using the program Repeatmasker [50]. Green: Arbitrarily selected 17mer from the L1 element. Although this 17mer appears very often in the genome in contrast to any randomly-chosen 17mer, the frequency density is significantly different from the selected ALU consensus 17mer.

COMBO-FISH probes are usually about 20 bp long, and must fit perfectly to the target region. For that reason, it was necessary to choose a part of the ALU consensus sequence which has as many targets in the genome as possible, but only in the ALU regions. With the help of special in-house software, a 17 bp probe was found (5’-3’) (***TAATCCCAGCACTTTGG***) binding to the complementary strand by Watson–Crick bonding, since it consists of purine and pyrimidine nucleotides. It was checked against the whole genome to determine whether the probe only binds in ALU regions. A map was computed which schematically shows this expected distribution of this ALU probe along the human chromosomes (Figure 2). The selected ALU probe sequence appears 385,924 times in the genome. The number of hits of the consensus sequence was 1,180,685. Of all the 17mer probe sequences, 99.9% are located within consensus sequences. Thus, the probe covers approximately 30% of the ALU elements—a proportion that is sufficient to represent the distribution of the ALU elements microscopically.

For comparison, another 17mer was arbitrarily selected from the L1-elements (***GGTGATTTCTGCATTTC***) and depicted in the same way (Figure 2). L1 belongs to the LINEs (long interspersed nuclear elements). The frequency density shows a completely different distribution, indicating that the frequency distributions are specific for any given 17mer sequence.

The oligonucleotide COMBO-FISH ALU probe carrying one fluorochrome (Alexa 568) at the 5′ end was manufactured commercially and hybridized according to the protocol described below. As a control, we also designed the most frequently occurring unique COMBO-FISH probe of the L1-elements according to the same procedure as for ALU sequences, and applied this probe together with the ALU COMBO-FISH probe to cell nuclei. The probe tags of ALU and L1 elements did not co-localize in the localization image (data not shown and will be published in detail elsewhere).

The successfully designed uniquely-binding COMBO-FISH ALU probe was applied on the breast cancer cell line SkBr3 cells. On widefield images, by visual inspection, the fluorescence results are comparable to other ALU-studies prepared by conventional microscopy [49].

### 2.3. Two-Color Localization Microscopy and ALU Clustering

Two-color localization microscopy was applied to further analyze the quality of the protocol and to simultaneously quantify the nano-structural arrangements of ALU repeats and heterochromatin (Figure 4). First, it was shown that the amount of co-localization between ALU and heterochromatin is negligibly low, as expected [51]. Furthermore, it was proofed that ALU sequence-dense regions are spatially associated but preclusive to labelled heterochromatin (Figure 4) [52].

Cluster analysis software was applied to the localization images [46,47]. The algorithm identifies “cluster-points” within the localization data according to user-defined parameters. These parameters are the minimum number of fluorescence signals within a defined circular region around each blinking event and the radius of this region. An event is identified as a “cluster-point” if at least as many points as defined as minimum points are in the predefined radius. The remaining points are identified as “noise-points”. The points are then divided into clusters. If two cluster-points have a smaller distance than the given radius, they belong to one cluster. In addition, all noise-points whose distance to a cluster-point is smaller than the radius also belong to the cluster. This cluster search algorithm is called density-based spatial clustering of applications with noise (DBSCAN) [53]. In this case, this allows the identification of ALU sequence-dense regions.

**Figure 3:**
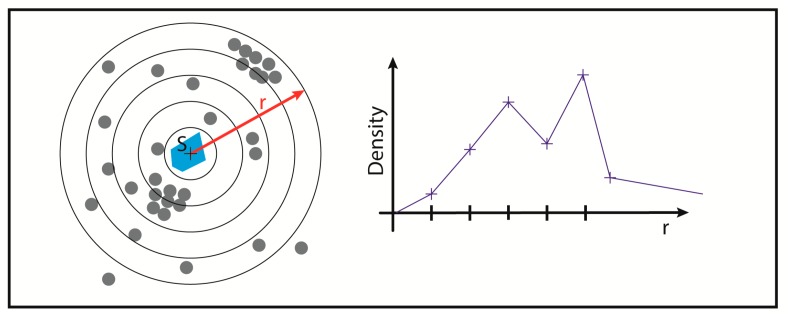
Density determination around ALU clusters. A cluster identified by density-based spatial clustering of applications with noise (DBSCAN) is shown in blue. Around the center of gravity S of this cluster, the density (in number of points per area) is now determined in concentric circular shells with a fixed thickness.

Here, a radius of 100 nm and minimum point number of 10 was used to identify “ALU cluster”, which means that there is a compact area of many repetitive ALU elements. After cluster recognition, the centers of the ALU clusters were computed with the Surveyor’s Area Formula [54]. These centers were used as the center points of increasing circle shells with the size of 10 nm. The heterochromatin density in these shells was computed (Figure 3) (heterochromatin density = number of heterochromatin points/area of the corresponding circle shell).

**Figure 4:**
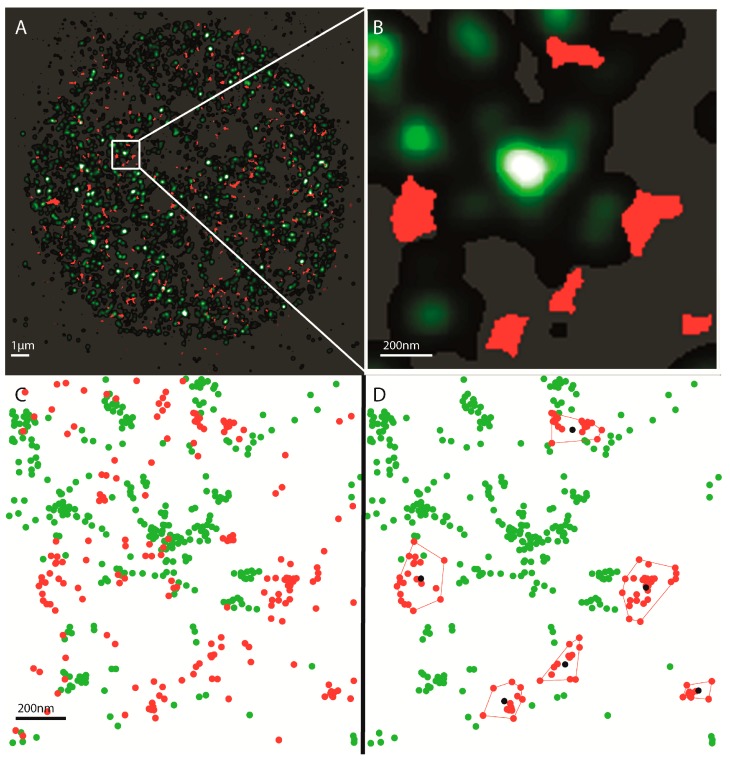
Image of the ALU clusters labelled by combinatorial oligonucleotide fluorescence in situ hybridization (COMBO-FISH) probes (red) and heterochromatin labelled by H3K9me3 antibodies (green). (**A**) Density image: Pixelsize = 10 nm/pixel, radius = 1000 nm, Gaussian filter *σ* = 50 nm; (**B**) Zoom of the marked region indicating the closed point patterns; (**C**) and (**D**) binary depiction of the same region shown in (**B**); (**C**) Every point corresponds to a single fluorophore; (**D**) ALU clusters and their barycentre (black) are shown.

**Figure 5:**
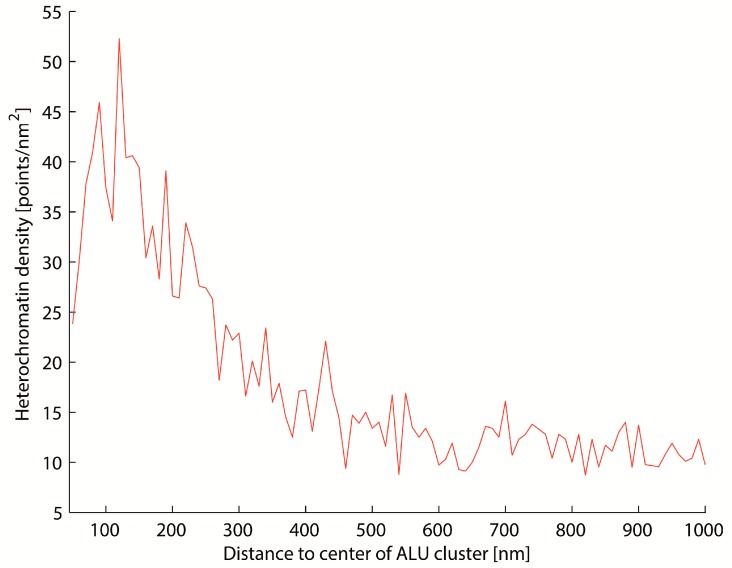
Mean heterochromatin density around ALU clusters for one cell. Approximately 200 ALU clusters were detected per cell. The density distribution for every ALU cluster was determined, and the mean value was computed.

**Figure 6:**
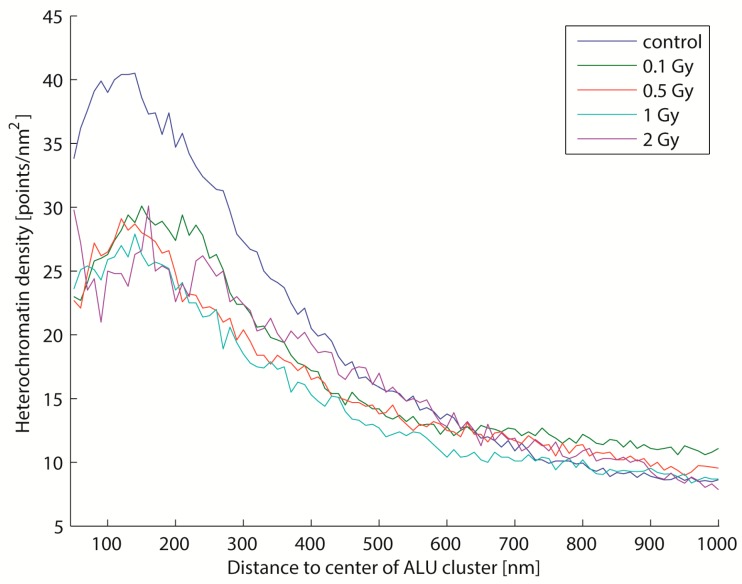
Mean heterochromatin density around ALU clusters after irradiation with different doses (about 200 ALU clusters were detected per cell, 30 to 40 cells were analysed per dose value). The density distribution for every ALU cluster was determined, and the mean values were computed. The data shows that irradiation influences the heterochromatin density around ALU clusters. A clear difference between irradiated and non-irradiated cells is visible, indicating a de-compaction. However, the underlying mechanism seems to be independent of the dose, since no considerable differences are visible between the irradiations (0.1 to 2 Gy).

The results show that ALU and heterochromatin are spatially associated but do not co-localize or intermingle (Figure 4, Figure 5 and Figure 6). Higher densities of heterochromatin are found within the near neighborhood of ALU clusters, but almost never in a co-localization, which indicates an intermingling arrangement. This corresponds to the results from other studies [49,52,55]. As an example, a typical density distribution for a cell nucleus is shown in Figure 5.

### 2.4. Biological Dosimetry by ALU Point Counting

**Figure 7:**
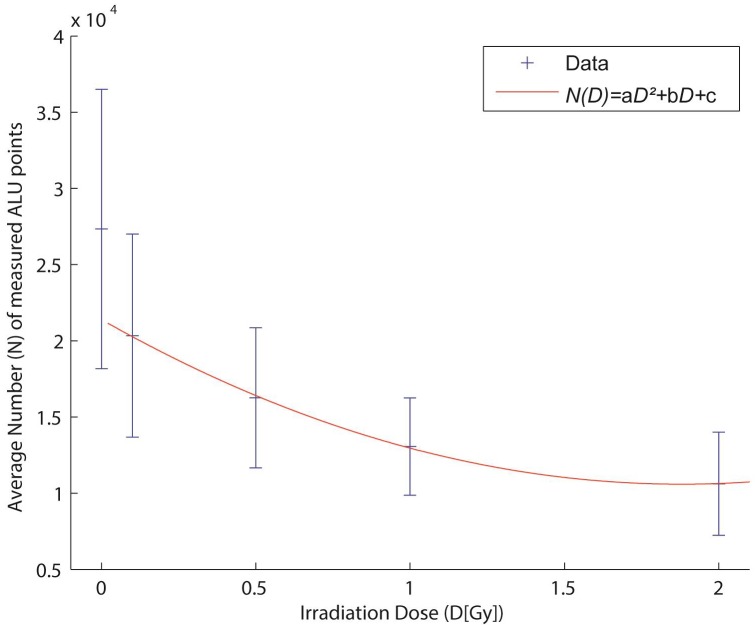
Average number of measured ALU elements relative to the radiation exposure dose as detected by specimen fixation 30 min after irradiation with 6 MeV photons. The measurement data is fitted by a linear-quadratic curve (*N(D) =* a*D*^2^ *+* b*D +* c) with the parameters a = 3054, b = −1.148 × 10^4^, c = 2.139 × 10^4^.

The combination of COMBO-FISH and localization microscopy allows the detailed analysis of the number of ALU elements in the nucleus. At a first glimpse, the measured number of points does not equal the real number of ALU consensus elements if one estimates the amount of points that could occur in the whole 3D nucleus from Figure 4. Hybridization and measurement efficiency reduce the amount of detected ALU oligonucleotide labels. Assuming a constant hybridization and measurement efficiency for all experiments, the absolute numbers detected and its dose-dependent decrease could be estimated to be proportional to the real values. This offers an opportunity for biological dosimetry (Figure 7). The number of ALU COMBO-FISH signals detected in a nucleus inversely follows the dose increase. By appropriate fitting, we were able to prepare a dose–effect curve with the following parameters:

*N*(*D*) = a*D*^2^ + b*D* + c

where *N* means the number of detected points (= ALU sequences), *D* the radiation dose, and a, b, c empirical constants of the fit (see the figure legend). The data show that irradiation may functionally or structurally (maybe by dimerization) influence the ALU sequences so that the accessibility of the probes is reduced.

Irradiation also changes the density distribution of heterochromatin around ALU clusters (Figure 6). The difference between the irradiated cells and the control cells is remarkable. It seems that irradiation changed the conformation of the chromatin structure and led to a decrease of heterochromatin density (de-compaction of the labelled sites) in the near-environment around the ALU cluster. This is in good agreement with recently published data [45,56].

## 3. Discussion

Usually, COMBO-FISH is used to specifically stain small units of the genome (e.g., a given centromere or a given gene of a chromosome) [21,22,23,57]. In this work, we used a uniquely specific COMBO-FISH probe to stain dispersed ALU consensus regions which can be found distributed all over the whole genome. By means of this probe, the distribution of ALU shows a kind of a “network”, as depicted by the pointillist visualization of localization microscopy. By recognizing ALU sequence-dense regions using a cluster search algorithm and analyzing the heterochromatin density, a characteristic preclusive association between ALU and heterochromatin was shown. It remains a challenge to find methods to analyze and mathematically quantify structures such as these and their changes. This would be necessary for analyses of effects on chromatin re-organization during and after environmental treatment such as irradiation. Novel theoretical procedures will be tasks of future projects. In this article, we have shown that the combination of labelling methods based on our newly-adapted protocols and super-resolution microscopy allows appropriate imaging for the quantification of structural parameters, and offers a variety of novel applications. Unfortunately, routine applications are mostly done on two dimensional (2D) optical sections, since 3D localization microscopy is still a challenge—especially for labelling stability and detection speed. Since the 2D data obtained represent a projection of a layer approximately 500 nm thick, they can be assumed to contain in part 3D information.

In this work, we were able to apply uniquely-binding purine–pyrimidine COMBO-FISH probes without heat treatment for specific chromatin labelling in cell nuclei, and to combine this technique with simultaneous immunostaining in the nuclei. Since this probe does not bind as a triple strand, not all structure-influencing steps could be completely dispensed (e.g., chemical denaturation with hydrochloric acid). Omitting heat denaturation avoids the most chromatin-destructive step, and represents a considerable improvement [6,7]. In principle, the COMBO-FISH protocol presented here might also be applied to standard FISH probes (e.g., BAC-probes) and thus combined with immunohistochemical staining. However, commercially-available FISH-probes are delivered in a ready-to-use buffer mix that is adapted to protocols including heat denaturation [5]. Moreover, the longer such a BAC-probe is, the more it requires a denaturation of a longer part of the DNA strand, which only seems to possible by heat or harsh chemical treatment [58].

Here, the stained cells were not exposed to such harsh structure-changing steps in comparison to standard methods using commercially-available probes. It is possible to get the full benefit of the improved resolution of localization microscopy with these methods, since nano-scaled structuring is mainly maintained in the native form. These localization data were evaluated using specially optimized analytical computer algorithms.

The mentioned results show a spatial arrangement of ALU consensus sequences and heterochromatin which has been so far impossible to investigate on the single cell level without high-resolution microscopy. At this point, we were able to microscopically confirm the hypothesis that heterochromatin and ALU do not co-localize, but may associate functionally in a certain way. We also gained some information about the structural connection of ALU and heterochromatin, which could contribute to a better understanding of chromatin interaction mechanisms after irradiation and during repair [52,59,60]. Since our major goal was methodological development, we used the SkBr3 cell line as an easy-to-handle but well-established cell line in breast cancer research [61]. Nevertheless, the conclusions drawn—especially by the application of the H3K9me3 antibody—should be verified by other cell lines (i.e., ideally, normal cells). This, as well as the study of our approach of biological dosimetry in different cell systems will be tasks of future investigations.

Although the dose-correlated density measurements have only been shown for one cell type, the reproducible sensitivity of the applied quantification indicates the potential of sensitive biological dosimetry, and therefore offers a broad spectrum of future investigations. However, further research seems to be necessary to understand the mechanisms behind the inverse correlation of ALU signals and dose. Whether there may be a pure methodological reason due to probe accessibility or a more complex reason based on biological mechanisms of chromatin modification and association, the reproducible dose–effect correlation offers alternative approaches to biological dosimetry beyond metaphase or micro-nuclei analyses.

## 4. Materials and Methods

In the following, the developed protocol for the combination of COMBO-FISH and immunostaining is described:

### 4.1. Staining Materials and Concentrations (Immunostaining and COMBO-FISH)

0.1M HCl in H_2_O; 1× Phosphate-buffered saline buffer (PBS) + MgCl_2_ (0.901 mM)/CaCl_2_ (0.493 mM); 0.05% Triton-X in 1× PBS + Mg/Ca; 2× Saline–sodium citrate buffer (SSC) (pH 7.4); 4% Formaldehyde solution (in 1× PBS + Mg/Ca); 2% Formaldehyde solution (in 1× PBS + Mg/Ca); Blocking solution (2% BSA in 1× PBS + Mg/Ca); Permeabilisation solution (0.2% Triton-X in 1× PBS+ Mg/Ca); 4′,6-Diamidino-2-phenylindole (DAPI)-solution (100–500 ng/mL in 1× PBS); 50% Formamide in 2× SSC; Object slides; Cover slips (1,5#) 24 × 24 mm; Fixogum; Embedding medium ProlongGold (ThermoFischer, Massachusetts, USA, ProLong^®^ Gold Antifade Mountant, P36930); COMBO-FISH probe solution (IBA life science, Goettingen, Germany, ALU sequence: Alexa Fluor^®^ 568-TAATCCCAGCACTTTGG; L1 sequence: Alexa Fluor^®^ 488-GAAATGCAAATCAAAAC): 200 ng probes in 20 µL Tris-HCl (pH 7.4); Primary Antibody: H3K9me3 (concentration: 1.4 mg/L, Abcam plc, Cambridge, UK, Anti-Histone H3 (tri methyl K9) antibody—ChIP Grade (ab8898)); Secondary Antibody: A488 (concentration: 4 mg/L, Abcam plc, Goat Anti-Rabbit IgG H&L (Alexa Fluor^®^ 488) (ab150077)); Humid heat chamber (37 °C); Shaker.

### 4.2. Handling Steps

The cells were grown on coverslips to about 80% confluency in the appropriate medium required for the cell type or cell line to be investigated. The cells were washed in 1× PBS + Mg/Ca for 5 min. After that, the cell specimen was fixed in 4% formaldehyde (in 1× PBS + Mg/Ca) for 10 min at 37 °C. After washing three times in 1× PBS + Mg/Ca for 5 min on a shaker, the cells were incubated in permeabilisation solution for 3 min on a shaker followed by additional washing three times in 1× PBS + Mg/Ca on a shaker for 5 min and incubation into the blocking solution for 30 min. Incubation with the primary antibody in a humidified chamber at 37 °C for 30 min and washing three times in 1× PBS + Mg/Ca on a shaker for 5 min was then followed by incubation with the secondary antibody in a humidified chamber at 37 °C for 30 min and washing three times in 1× PBS + Mg/Ca on a shaker for 5 min. The specimen was again fixed in 2% formaldehyde solution (in 1× PBS + Mg/Ca) at 37 °C for 10 min and washed three times in 1× PBS + Mg/Ca on a shaker for 5 min. The cells were incubated in 0.1 M HCl for 10 min and washed three times in 0.05% Triton-X in 1× PBS + Mg/Ca on a shaker for 5 min. After two steps of incubation in 2× SSC (5 min) and in 50% formamide in 2× SSC (30 min), 20 µL of COMBO-FISH probe solution was given on a slide. The oligonucleotides in the probe solution have to be determined by computer search and synthesized with high purity (for details of computer search, see above). The coverslips with cells were put on the slide and sealed with rubber cement (Fixogum) for incubation in a humidified chamber at 37 °C for 24 h. After removing the Fixogum and washing three times in 2× SSC at 37 °C on a shaker for 5 min, the specimen was incubated in 1× PBS + Mg/Ca for 5 min, and the chromatin was counterstained with DAPI for 5 min. Finally, the specimen was washed twice in 1× PBS + Mg/Ca on a shaker for 5 min and embedded in 15 µL ProlongGold (ThermoFischer) embedding medium on a new slide. After sealing with nail polish, the specimen can be stored at 4 °C and should be used for localization microscopy within the next fortnight.

### 4.3. Microscopy Setup

**Figure 8:**
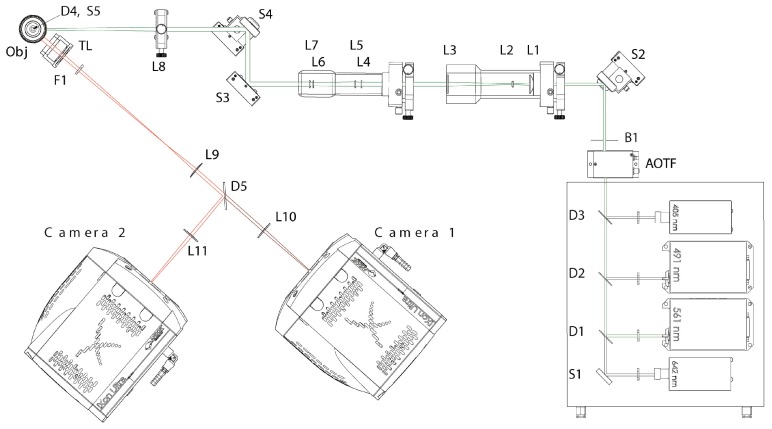
Schematic representation of the microscopic setup (see text for details).

The microscope (Figure 8) was built at the Light Microscopy Facility of the German Cancer Research Center in Heidelberg. Four lasers with wavelengths of 405, 491, 561, and 642 nm are available. The mirror S1 and the dichroic mirrors D1, D2, and D3 bring the four lasers to a common optical axis. An acousto-optic tunable filter (AOTF) is used to select the laser wavelength and set the intensity. Since—in contrast to adjustable filters—no mechanical movement is required, the beam path is stable when changing the wavelength and changing the intensity. In the case of measurements of the same sample with different lasers, this reduces the displacements between the images. Lenses L1, L2, and L3 expand the beam since the subsequent lens system for homogenizing the beam intensity (consisting of lenses L4, L5, L6, and L7) requires a laser beam of fixed diameter. Homogenization is important for localization microscopy because the reversible photobleaching is dependent on the laser intensity, and therefore a homogeneous illumination is assumed. Various mirrors (S) serve to change the direction of the beam. Through the focusing lens, L8, and the mirror, S5, the beam is directed into the objective by the dichroic mirror, D4. A 100× oil immersion lens with a numerical aperture of NA = 1.46 is used. The light captured by the lens is imaged onto the EmCCD-camera by the dichroic mirror, D4, a tube lens, TL, a blocking filter, F1, the lens system (L9 and L10 or L9 and L11), and the dichroic filter, D5. The system used here did not yet have the tube lens, the second camera, or the dichroic mirror D5. Here the laser with the wavelength 491 nm and 561 nm were used. In localization mode, the laser intensity was 3 KW/cm^2^ (491 nm) and 5 kW/cm² (561 nm). The exposure time was 100 ms per frame. Two thousand frames were captured in each channel.

## 5. Conclusions

The application of localization microscopy offers new insights into chromatin conformation on the nanoscale. Quantitative analyses of chromatin conformation changes also require appropriate techniques of nano-probing since voluminous probes might impact the native conformational organization. In order to combine different nano-probing techniques like antibody-labelling or oligonucleotide sequence labelling we developed a low temperature protocol that allows COMBO-FISH labelling and immune-staining simultaneously. Based on this development we were able to show dose dependent conformation changes of ALU sequence clusters and heterochromatin. The results show the new approach towards biological dosimetry using conformational changes as dose related parameter. In contrast to well established techniques of biological dosimetry, this approach, although requiring super-resolution microscopy has the advantage that the number of cells necessary for analyses can be reduced.
